# Should Robotic Surgery Simulation Be Introduced in the Core Surgical Training Curriculum?

**DOI:** 10.3389/fsurg.2021.595203

**Published:** 2021-03-10

**Authors:** Kunal Bhakhri, K. Harrison-Phipps, Leanne Harling, T. Routledge

**Affiliations:** Guy's and St Thomas' NHS Foundation Trust, London, United Kingdom

**Keywords:** laparoscopic surgery, core surgical training, video assisted thoracoscopic surgery, surgical simulation, robotic simulation

## Abstract

**Introduction:** The focus of this research is to qualitatively analyse the literature and address the knowledge gap between robotic surgery simulation (RoSS) and core surgical training curriculum. It will compare the effectiveness and the benefits of using robotic simulators in training as compared to the current standard training methods.

**Materials and Methods:** A qualitative research of literature was carried out with the use of critical analysis formatting to expand the search. The inclusion criteria entailed selecting academic resources that focused on Robotic Surgery Simulation (RoSS) and core surgical curriculum. The Online databases used in the search took into account information retrieval from stakeholders.

**Evidence Synthesis:** In this article, we compiled and scrutinized the available relevant literature comparing performance assessments, surgical skills transfer and assessment tools between robotic surgery simulation (RoSS) and current training platforms in open and minimal access surgery. Data that has been published underpins the authenticity of robotic Surgery Simulation (RoSS), based on a combination of observational evaluation and simulation scores.

**Conclusion:** The introduction of robotic surgery simulation (RoSS) has the potential to bring major improvements in the surgical training curriculum. RoSS platforms are more robust in terms of ensuring rapid surgical skills transfer/ acquisition, assessment is standardized, unbiased and the training covers non-technical skills aspects.

## Introduction and Background to the Topic

While robotics is widely applied in different fields, the medical use of this equipment has been on the rise. According to Arellano and González (1), robotics is used in urology and other medical disciplines (2). However, robotic training and simulation still trail to adopt this form of surgical technology. There is an immense need to review surgical training and propose a training model, curriculum, as well as assessment to training students' surgeons before they commence surgical practice. This is essential in improving surgeons' skills, mastery, and proficiency to reduce the possibility of patient harm. Over the years, the medical field has witnessed many innovations that sanctioned rapid growth and change. Medical innovations take different forms, such as pharmaceutical breakthroughs, new knowledge, advanced technology, and techniques. Several pieces of literature have discussed the use of robotic surgery as a pivotal tool in the overall training. Despite the widespread use of robotics in the medical field, the application of this important equipment has been underestimated in the training.

The robotics systems in surgery have grown exponentially and further become a sophisticated type of minimal access surgery. The central aspect is how laparoscopic and thoracoscopic surgery can be accomplished through robotic training, whereby a supervisor steps aside and allows the trainee to undergo the complex and sophisticated steps. Although the trainer might be in control, the robotics surgery can be performed within the shortest time. According to Mirheydar et al. (3), the use of traditional laparoscopy is applied to various reconstructive challenges that are witnessed as long periods are needed in acquiring the required confidence and skills in the trainees. However, the robotic-assisted laparoscopy and thoracoscopic surgery (RALTS) can shorten the access of necessary skills by the trainees. Sridhar et al. (4) go further to explain that the robotic-assisted laparoscopy and thoracoscopic surgery is effective and safe when applied to children. For instance, Sridhar et al. (4) claim that the introduction of Robotic surgery in the hospital in November 2014 in Sunnyvale, CA. The U.S.A and the number of surgeries performed were higher than the use of conventional surgeries. Using RALTS, the surgeon performed 154 surgeries; hence, revealing the importance of ingrained in the robotics surgery.

Several scholars have documented that training using robotics surgery is essential since many programs that aid the training to gain the necessary training in the UK (5). Some of these trainings are the intuitive UK, Multispecialty Robotics Training Center, and the da Vinci surgical systems and robotics programs. These programs allow the student's surgeon to conduct numerous surgical procedures that could not have been possible in case traditional forms are used. As such, the trainees are granted more time even before they touch the patient (5). The available programmes provide the necessary skills and exercises that can be personalized in different surgical specialties.

The use of robotics training systems should be applied in the early years of training. Since surgical training takes approximately 8 years, whereby 2 years are designed for the training of core principles of surgery while 6 years are designed for specialty training, Robotic training is applied to all various specialties such as console docking and team learning (5). Training may entail console training, which incorporates supervised robotic operating, dry and wet lab simulations. Advanced programs entail the use of diathermy, suturing, and excision. Normally patient side training entails involves basic laparoscopic skills, robot docking, procedure-specific port placement, pneumoperitoneum, and patient positioning.

The training also entails virtual reality simulators, which take a basic console control system when carrying out complex tasks. Currently, there are five virtual reality simulators, which are available for robotic surgical training. These include the robotics mentor, da Vinci skills simulator, SimSurgery Norway, or SEP Robot, dV-Trainer, and Robotic Surgical Simulator (6). The use of virtual simulators in training has been reportedly efficient based on laparoscopic outcomes since they do not inflict injuries. These training simulators are reportedly to have immense strengths as opposed to weaknesses. Providing early training enables the trainees to familiarize themselves and gain the required expertise within a short period.

One of the widely recognized colleges that has applied robotics and surgical simulations is the Kings College Urology, which has been among the leading in providing robotics-assisted surgery in urology. Morris (7) observes that Kings College Urology is the only leading Robotic Surgery that has successfully applied robotics systems in its various works. In this case, the clinical fellowship organized by the college has widely been recognized by the European Association of Urology. The Hospital is reportedly been treating over 6,500 patients with prostate cancer and it is perceived as among the busiest Prostrate Cancer Service provider in the world.

### Research Question

Should Robotic surgery simulation be introduced early in the surgical curriculum?

### The Rationale for the Research Question

To understand and approach this evidence-based practice, the proposed research question above was essential in guiding the literature search. This evidence-based paper recognizes the fact that robotics surgery has become a growing and sophisticated issue in the surgical field. As such, the surgeons perceive the robotic system as a practice, which is essential in providing control, flexibility, and precision. This system can also assist in performing different types of procedures and further provide real-time feedback to the surgeon; hence, making doctors conduct operations on the patients with confidence. In this case, the robotics systems in the operating rooms enable the surgeons to make the decisions that affect the lives of their patients positively (8). Furthermore, these systems are designed to promote intelligent surgery by developing goals that aim at reducing variability and enhance better outcomes to the overall surgical care in the medical industry.

Remarkably, the Royal College of Surgeons training curriculum has not included robotic surgery in training the surgeons. Since there are currently many options to offer robotics services that can be applied training of student surgeons, the application of these systems will promote and expand their skills, knowledge, and expertise (8). Therefore, this evidence-based assessment focuses on available evidence on the impact of providing robotics surgery at early training as opposed to the current practice whereby trainees are compelled to use it at the end of their training or during consultancy.

## Literature Search

**Figure 1:**
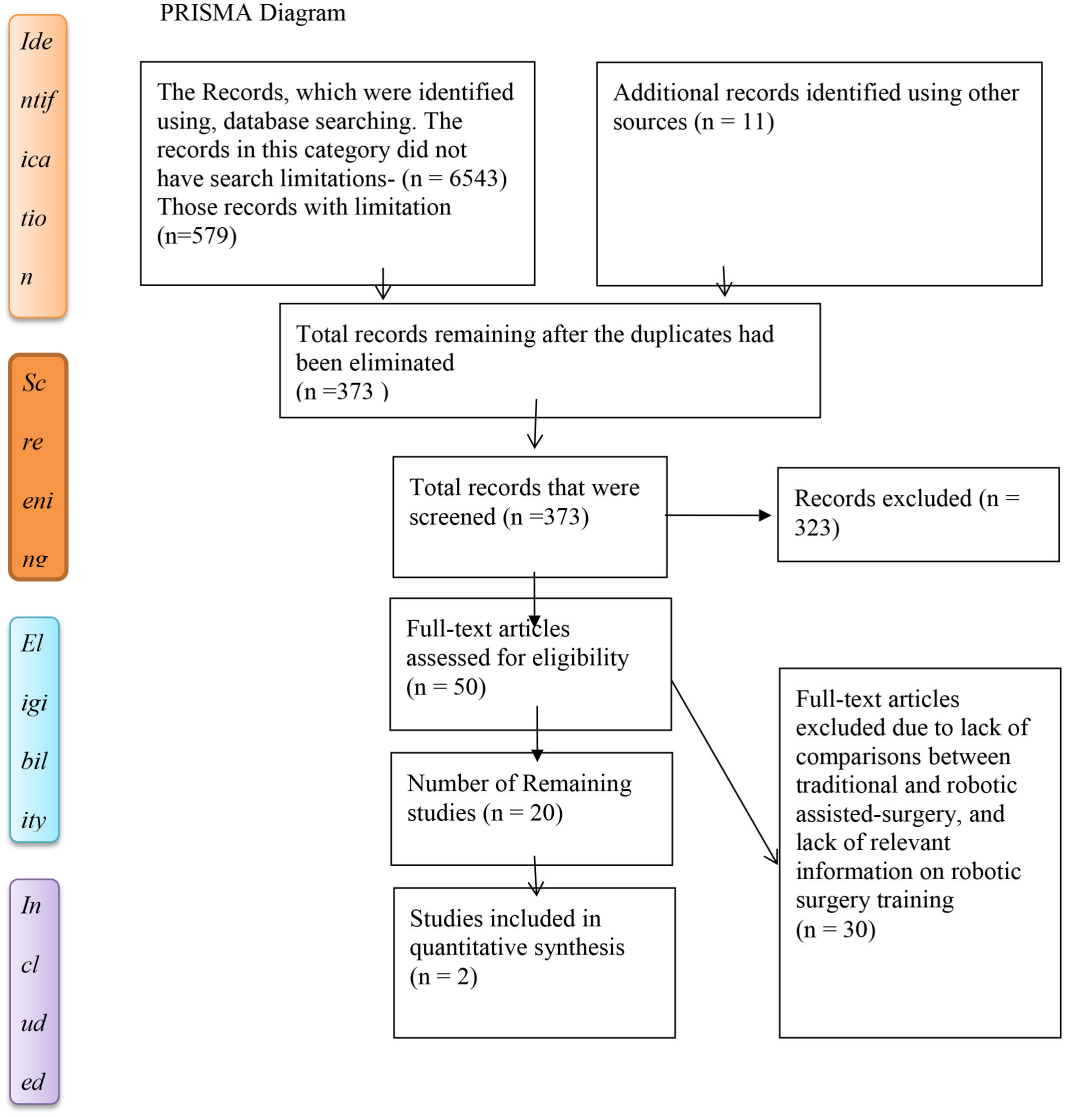
PRISMA flow diagram aid used as a tool to include studies in the qualitative analysis.

The success of this assessment was based on the tools employed for the search of relevant literature materials. In this regard, the search was based on terms that were used in obtaining the relevant literature for the assessment, a presentation of the PRISMA diagram, and rationale for selected papers (Figure 1).

### Inclusion Criteria

The main model used was the Robotic Surgical Simulator (RoSS) (9). Based on the search strategy, the outcomes were reduced time of surgery, especially in urology, which was very effective as opposed to traditional ones. The effectiveness of the surgical procedures enhanced patient satisfaction and high educator ratings since immense skills and knowledge for the trainees were enhanced.

### Exclusion Criterion

Didactic only training and *in-situ* training/working with a real patient were considered (10). The period for the training eliminated by the exclusion criteria resulted in a narrow search and hence the time taken may be a topic to be investigated.

### Rationale for Databases

Since this assessment was primarily based on secondary sources, the uses of various databases to extract various sources were essential (11). Obtaining literature is pivotal in medical research. The aim is to include accessing available evidence concerning the question raised above in this assessment. The literature revolved around the question “Should Robotic surgery simulation be introduced early in the surgical curriculum?” The databases employed in this review were since they included unbiased and pre-defined search strategy (12) (Table 1).

**Table 1:** PICO analysis tool for framing the research question.

**Patient or Population or Problem**	**Intervention or Exposure**	**Comparison or Control (if applicable)**	**Outcomes or Effects**
Robotics Training	Simulation-based models in robotics surgical training	N/A	Improved skills, experience, knowledge, and expertise
Robotics * Training curriculum* Robotics systems* Thoracoscopic* Laparoscopic Surgical Training* Robotic simulation* robotics surgery* robotic-assisted* surgeons* Urology* Console training Students surgeon Training model	The interventions entailed the applications of various models, in particular, Robotic Surgical Simulator (RoSS)		Reduced time of surgery

CINAHL, PubMed, Cochrane Library, TRIP, and SCOPUS were among the main databases that were used in this search to retrieve the relevant information. In this regard, these databases provided peer-reviewed sources, information including systematic reviews performed in the robotics surgery, training, and gold standard sources that closely incline to the subject under investigation. In this case, CINAHL yielded 9372283, PubMed 3289929, Cochrane Library 2134722, TRIP 534228, and SCOPUS 421272. Most of these entailed the highest level of evidence, whereby they consisted of systematic reviews, and randomized controlled trials (13). In this case, CINAHL yielded the highest level and the least was Scopus. These databases can be justified since they had extensive collections of articles and many reputable sources. In this regard, reputable sources imply books, articles, and papers that were written by highly recognized authors with respected academic and professional background. Similarly, these databases had an up-to-date research articles, books, and other materials, which were rarely available in other databases (13). Collectively, the results obtained for CINAHL, PubMed, Cochrane Library, TRIP, and SCOPUS after searching the key words were 64143734. Therefore, these databases yielded a high number of results as compared to other search engines.

All the databases used in this category had extensive articles that directly relate to the subject matter of healthcare research, health education, robotics-assisted surgery, and nursing fields. These fields are essential in providing the necessary information required for building upon this topic. Databases such as Scopus, CIHNAL, PubMed/Medline, and Cochrane provide bibliographical databases and a wide variety of sources that a researcher can select from Moher et al. (13). The main work of Boolean Operators is excluding keywords in the search database to have a more focused result. This tool assisted me excluding several papers, which lacked relevant information concerning the topic under study. ASSIA, Web of Knowledge (Science & Social Science), whereby attempts to use these databases were made although they were unsuccessful since the institution had not registered with them.

Healthcare Management Information Consortium (HMIC) was also considered as a valuable database that could provide relevant information under the subject of investigation. This site provides information concerning various education processes. The information obtained from this search could yield the required information that can influence policy to adopt training programs that allow the institutions to use robotic-assisted surgery at the early stages of training. In essence, HMIC can provide much-needed information for healthcare managers and administrators to make informed decisions about robotics in the healthcare industry. Various search terms in these databases yielded several results; hence, this site was considered as an invaluable source of information.

Another important database was EBSCOhost, which is among the world's largest educational database. These databases provide a wide range of information ranging from conference papers, reports, articles, journals as well as indexed materials through the formal reviews. In this regard, EBSCOhost database was considered an important tool for the search strategy in which institutional learning provided easy access through its login. Although the results of searches were not straightforward, the articles and journals obtained from this site closely correlated with the subject under study. The total searches obtained from this database were 1223. After applying exclusion criteria, I remained with a few papers that were used in this assessment.

U.S National Library of Medicine Database of Clinical Trials is another important database that considered in the search strategy is the U.S National Library of Medicine Database of Clinical Trials, which is perceived as an excellent source with the required literature on the subject under study. Since it was challenging to apply the complete collection of search terms in other databases, this provided an opportunity for complete use databases and yielded several results that were required for this assessment. This has been demonstrated in the figure below.

Biomed Central is a search performed on Biomed Central provided several results, which were 156224 in total. However, the challenge with this database was the lack of filters to limit the number to materials with the required information under the topic of study. Therefore, the Biomed Central was excluded from the overall search. NICE Guidelines Site NICE was an essential part of the search strategy that includes relevant training information on the medical training of professionals. This site provides information concerning the training of professionals in the medical field. Nonetheless, this demands a complex search of materials, which ended up yielding no results. In this case, this search was excluded.

### Restricting Searches per Database

Finding relevant information was essential and demanded the researcher to restrict the search results to the required ones as follows.

#### Cochrane Library

Cochrane Library is among databases that yielded high results based on the research question. Some of these searches revolved around technological researches (13).

#### PubMed Database

The use of this search engine based on Boolean operators yielded many results; hence, there was a need for filtering some of these results to obtain the relevant materials on the subject under study. Boolean operators enabled the researcher to direct and focus the research to more specific paper. This criterion excluded the sources that were not relevant in answering the research question. Some of the search results revolved around the following topics, “Surgical robotics and laparoscopic training drills,” “Implementing a robotics curriculum at an academic general surgery training program,” and “Human-centered robotics applied to gait training and assessment.” The topics of articles and other materials obtained in this database were examined to establish whether the authors were reputable or not. Equally, the relevance of the topics was tested. Most papers on this site were found to be suitable for the research question. In this case, the application of filters was essential in obtaining a manageable number of papers in specific areas of research.

#### CIHNAL

This search strategy provided the required number of sources, which were relevant. Restrictions or filters were not applied since most of the searches that were found were relevant.

#### TRIP

This search database was essential since it provided a variety of unmanageable materials. In this case, it produced ~28112 results. The need for filters was essential to restrict the results to a few papers, which were relevant to the research question. After keying in the search words, I obtained almost 534228 results, which is the total of the key words I used for searching. This site yielded high number of results as shown in the Appendix 1.

#### Scopus

Just like other databases named above, Scopus was essential as it yielded high results of ~421272. This site also provided limitations that allowed me to focus on a few papers that address the subject of the paper.

## Critical Appraisal

### The Rationale for Critical Appraisal Tool

The critical appraisal tool used in this research has been designed to assist an evidence-based approach. The instrument used in this research emphasized study aims, methods used in the research, and the sample selections. The selected literature emphasizes on robotic training and simulation in surgery; hence, revealing the numerous debates among the educational professionals. There have been many literacy works that discuss the use of simulators, particularly how to validate and prove the fidelity and reliability of simulators. In the search, only 40 pieces of literature out of the 373 discussed simulators to be integrated into the curriculum of surgical training. Markedly, 55 literature review works explored the implementation of simulators when evaluating students' surgical skills. Within the studies, 34 had 40 trainees with five pieces of literature, having more than one hundred students. The principal objective of integrating simulation in the curriculum of surgical training is to allow trainees to acquire relevant technical skills according to the required training levels.

This is achieved in a training environment that is safe from the perspective of both patients and students. Surgical training in simulation is significant to surgical students and during monetarization of student's progress until they have acquired substantial skills minus putting the lives of patients at risk. This requires continuous assessment and training. The traditional training employed for surgical students involved cross-examination of feedback from supervisors and students' logbook records after a given timeframe. A limitation of the records is that they are not excellent markers of student's expertise (12).

The feedback written by a supervisor indicates the overall performance but does not show the technical acquired by the student. Hence, these kinds of assessments are mostly subjective and depend on several variables; for example, the condition of a patient, the atmosphere in the theater, and the condition in a hospital. In the area of plastic surgery, it has been proved that candidates for the HST program got an excellent performance in the whole 6 tasks such as arterial anastomosis, lipoma excision, tendon repair, laceration repair, sebaceous cyst excision, and Z-plasty. Compared to students that did not. Therefore, the Objective Structured Assessment of Technical Skills (OSATS) stands to be the most preferred technique for assessing surgical skills (12). It can be used for procedures in open surgery combined with inanimate models, for example, a bowel anastomosis, sebaceous cyst, or excision of lipoma, closure, and laparotomy incision. For every station, there is an expert surgeon to assess concerning the area of specialty. There is simultaneous operation of the stations during a period.

### Assumptions and Biases

The main assumption revolves around curriculum implementation that is required in the acquisition of skills within a simulated environment that can be transferred to an operative environment. If the assumption is true simulation-based training thus becomes a significant factor to produce expert surgeons and hence an excellent patient safety and care. In the last years, the techniques employed in observational evaluation, for example, the OSATS. The technique is a good methodology in the assessment of surgical skills. Despite the introduction of simulation technology in medicine, it is yet to be fully integrated into the curriculum of students' surgeons, especially when assessing open skills (9). Therefore, it is important to question the reason for this trend. Many works of literature have discussed the validity of the simulators. Since the introduction of the simulation technology, patient safety, and care has improved concerning reproducibility and fidelity. Lack of literature on the integration of simulators concerns data paucity when there is a need for translation of simulation-based pieces of training to fit patients' settings.

### Overall Evaluation/Synthesis Based on Critical Appraisal and Biases

Works of literature in this paper identifies studies that discussed virtual reality (in simulation) to produce metrics that combine errors systems of assigning scores to performance assessment. This technique is sensible when the evaluation of surgical skills is considered. Different data that have been published underpins the authenticity of simulators. Even though this evidence suggests only a few literacies, work highlights that the curriculum for surgical training will be integrated with simulators. The literature search employed in this paper was based on the English Language only. In general, only 12 of the identified works of literature discussed the integration of simulators in the surgical curriculum. The study population used lasted a sufficient period with the use of simulators in pieces of teaching and evaluation of skills. Only five studies identified the assessment of open skills based on observational techniques or OSATS. Only two literature studies talked about laparoscopic skills, while three literature studies combined observational evaluation and simulator's scores (14). However, single literature evaluated students' performance by employing different evaluation tools identified as OSATS, analysis of the end products, and ICSD. Besides, another literature study talks about endoscopic skills by combining simulator and the GAGE- based scores.

The above pieces of literature designed an intensive session for incoming or new residents to improve students' technical skills at the beginning of the program. The studies evaluated open skills in the technical fields employing observational techniques. As per laparoscopic skills, a single study employed computer-generated algorithms or metrics while the other literature employed the FLS scoring system. According to Fernandez et al. (15), performances of new residents increased after the nine-week course elapsed. Besides, for the previous study, a course in boot camp took three days, and the recorded performances never had substantial differences than for a controlled category of the study population. For simulation lab settings, only a single study evaluated students' performances then evaluation in OR settings. Upon simulator training to measure proficiency, surgery students performed laparoscopic cholecystectomy with their supervisors present, and a video record was used to record performance. The video recording was evaluated by employing observational tools.

However, this single formed literature that reported active inclusion of simulator-based training in surgery then translated into clinical practice. Therefore, it is evident that surgical skills evaluation in the lab environment is more robust. Despite the finding, a question arises whether the simulation-based skills training can be practiced within a clinical environment. This forms a topic for future research. A review done by Buckley et al. (14) showed that the program affects positively real-time operations. Also, a predefined score used to evaluate performance for the OR despite the quantifiable guidelines, for example, hand dominance, smooth movements, and ergonomics based on simulators' measures.

### Application to Practice

The evidence obtained in the literature reviews provided much-needed information for the implementation of the programmes in the medical field. Based on the literature provided above, robotics-assisted surgery can be applied in training with the supervision of the instructors.

## Reflection and Rationale on the Implications for Your Practice

The results of the literary works in this assessment have been scientifically justified as essential in promoting efficiency in the medical field. The literature has also revealed that robotics-assisted surgery promotes the faster acquisition of skills and expertise as opposed to traditional teaching methods that take a long time. However, several authors have cited various challenges with simulation exercises, whereby students may not receive the feedback as compared to the human instructional methods (14).

## Conclusion

The application of robotics in the healthcare industry has trailed behind, yet they have been employed in other industries. Robotics simulation can be applied surgical procedures mainly urology. Based on the research, application of robotics in the healthcare industry, in particular, surgical training has immense benefits to the students as it promotes and improves the acquisition of surgeons' skills, mastery, and proficiency to reduce the possibility of patient harm. Cocchia (12) provides that there is a need to review surgical training and propose a training model, curriculum, as well as assessment to training students' surgeons before they commence surgical practice. Several pieces of literature have discussed the use of robotic surgery as a pivotal tool in the overall training

## Data Availability Statement

The raw data supporting the conclusions of this article will be made available by the authors, without undue reservation.

## Author Contributions

KB: lead author, has undertaking a thesis in robotic surgery simulation for core surgical trainees. TR: extensively involved in robotic surgery training with an accredited programme, reviewer of article. KH-P: article review, 20 + years clinical experience in thoracic surgery, offering key insights into development of simulation development. LH: research into education and training with insight into analysis perspective on this research article. All authors contributed to the article and approved the submitted version.

## Conflict of Interest

The authors declare that the research was conducted in the absence of any commercial or financial relationships that could be construed as a potential conflict of interest.

## Appendix

**Appendix 1:** Results of search strategy.

**#**	**Search Term(s)**	**Cochrane**	**PubMed**	**TRIP**	**CIHNAL**	**Scopus**	**ERIC**	**Total**
1	Robotics	1823516	334932	142,675	900,254,342	322423	1223	2624765
2	Training	345,624	2,345,624	322423	232323	23323	2322	3,452,249
3	Curriculum	124288	345223	132443	2756	34234	5232	180226
4	Simulation	25	89	322	321	3121	2311	8459
5	Models	2001	3242	2342	23223	2321	32212	62861
6	Surgery	11	2322	2322	12232	43534	3232	45733
7	Thoracoscopic	32423	212	423	2323	13323	12232	64,345
8	Laparoscopic	211	1213	8621	421	3221	1217	9646
9	Surgical Training*	167	2368	2133	20221	21122	4	4273
10	Robotic simulation*	354	43454	334	125876	255682	193	755655
11	Robotics surgery*	2334	22322	8987	687	12543	102	24866
12	Robotic-assisted*	23232	3222	21122	2672	1254	1231	54293
13	Surgeons* Urology*	27232	31322	38	1232	234	16	59044
14	Surgeon training model	2324	234	2160	7282	2558	221	13759
15	Console training students	124	2324	1,422	42722	211	3221	43194
